# Lived experiences of patients and families with decentralised drug-resistant tuberculosis care in the Eastern Cape, South Africa

**DOI:** 10.4102/phcfm.v15i1.4255

**Published:** 2023-12-22

**Authors:** Joshua O. Iruedo, Michael K. Pather

**Affiliations:** 1 Department of Family Medicine and Primary Health Care, Faculty of Medicine and Health Sciences, Stellenbosch University, Cape Town, South Africa

**Keywords:** DR-TB, HIV, decentralised community care, OR Tambo district, South Africa

## Abstract

**Background:**

South Africa adopted the decentralised Drug Resistant Tuberculosis (DR-TB) care model in 2011 with a view of improving clinical outcomes.

**Aim:**

This study explores the experiences and perceptions of patients and family members on the effectiveness of a decentralised community DR-TB care model in the Oliver Reginald Kaizana (OR) Tambo district municipality of the Eastern Cape, South Africa.

**Method:**

In this phenomenological qualitative research design, a semi-structured interview with prompts was conducted on 30 participants (15 patients and 15 family members). Framework approach to thematic content analysis was adopted for qualitative data analysis.

**Results:**

Four themes emerged from the patients’ interviews: adequate knowledge of DR-TB and its transmission, fear of death and isolation, long travel distances, and exorbitant transportation cost. A ‘ready’ health system influenced the effectiveness of community DR-TB management, while interviews with family members yielded five themes: misconceptions about DR-TB, rapid diagnosis and adherence counselling, long travel distances, activated healthcare workers, and little role of traditional healer.

**Conclusion:**

A perceived effectiveness of a community DR-TB care model in the OR Tambo district was demonstrated through the quality and comprehensiveness of care rendered by a ‘ready’ health system with activated health care workers (HCWs) who provided robust support and adequate knowledge of DR-TB and its treatment/side effects. However, misconceptions about DR-TB, long travel distances to treatment facilities, high cost of transportation and stigma remained challenging for most patients and family members.

**Contribution:**

This study provides insight into the lived experiences of a decentralised community DR-TB care model in the OR Tambo district in 2020.

## Background

Drug Resistant Tuberculosis (DR-TB) is TB that is resistant to one or more anti-TB drugs, and continues to threaten global efforts towards eradicating TB.^1^ With about 500 000 cases diagnosed annually, and only about 40% notified, of which 32% are placed on appropriate treatment, the burden of DR-TB on patients, communities, and health system remains significant.^1^ This under-diagnosis and under-treatment of DR-TB continue to foster the transmission of resistant strains within the community.^1^ Globally, while there was an estimated 450 000 incident multidrug resistant tuberculosis (MDR-TB) cases in 2021, with 3.6% seen among new TB patients and 18.0% among previously treated patients, an estimated 191 000 people died as a result of DR-TB.^2^

South Africa remains a TB high burden country with a high incidence of MDR-TB and Rifampicin Resistant Tuberculosis (RR-TB) of 37 per 100 000 populations per year.^3^ With an estimated 30 000 incident cases and 18 734 notified cases of DR-TB in 2014, only 62% commenced DR-TB treatment.^4^ Latest figures of estimated incident cases of MDR-TB stand at 21 000 in 2021 following a scale-up in the availability of drug susceptibility testing (DST).^2^ Although DST has improved incrementally over the decade from 41% in 2014^4^ to reach 71% among all diagnosed TB cases in 2021,^5^ there has been a gap in treatment initiation. This is attributed to availability of beds at centralised TB hospitals,^6^ high early mortality especially among patients with human immunodeficiency virus (HIV) co-infection, stigma, programmatic challenges such as the need for better counselling at multiple levels, as well as socioeconomic and personal challenges.^7,8,9^

Effective management of DR-TB should incorporate not only the patients’ medical treatment, but also pay attention to psychosocial components of care.^10^ An integrated patient-centred approach to TB care is one of the three major pillars of the END TB Strategy developed by the World Health Organization (WHO) aiming to “end the global TB epidemic” by 2035.^10^ One of the underlining principles of this strategy is the protection and promotion of human rights, ethics, and equity.^10^ Patient centred care has been defined as care that is respectful of, and responsive to the individual patient preferences, values, needs, and ensuring that these serves as a guide to all clinical decision making.^11^ The term people-centred care which removes the label of sickness^12^ from the individual is preferred and involves rapid diagnosis of TB, good communication, staff training and health education, support (including psychosocial, nutritional, financial and transportation), decentralised care for treatment and prevention of TB, as well as collaborative health services.^10^ The other components of the END TB Strategy include broader policies and supportive services, including Universal Health Coverage (UHC), community engagement, intensified research and innovation.^11^

A decentralised community DR-TB care model may help to resolve some of the challenges currently facing a centralised care model with its shortcomings of unavailability of admission beds, unnecessary delays in treatment initiation, a feeling of isolation from being ‘locked down’ in centralised facilities, increased cost of in-patient care, and absence of social support structures.^6,13^ However, there is a concern about decentralised care given that DR-TB is an infectious disease with potential to spread within the community if adequate care is not given.^14^ This perception has led to stigmatisation of those affected who are mostly isolated and excluded from the community.^14^ For patients with DR-TB to have a good quality of life, the World Health Organization (WHO) recommends patient centred care, patient education, comprehensive care and support.^14^

Drug Resistant Tuberculosis is often seen as a problem of poor adherence to treatment. This reductionist view of non-adherence without exploring the underlying reasons poses ethical challenges and possible human rights violations.^15^ A previous study on DR-TB in Oliver Reginald (OR) Tambo district, Eastern Cape province, adopted a quantitative research design which does not allow for understanding of the illness experiences of patients.^6^ This study bridges this gap by exploring the lived experiences of patients and families on the effectiveness of a decentralised community DR-TB care model.

## Methods

### Study design

A phenomenological qualitative study that explored the lived experiences and perceptions of patients and families was adopted using semi-structured interviews with prompts, to look into improving our understanding of issues related to the management of DR-TB in the community.

### Study setting

**FIGURE 1:**
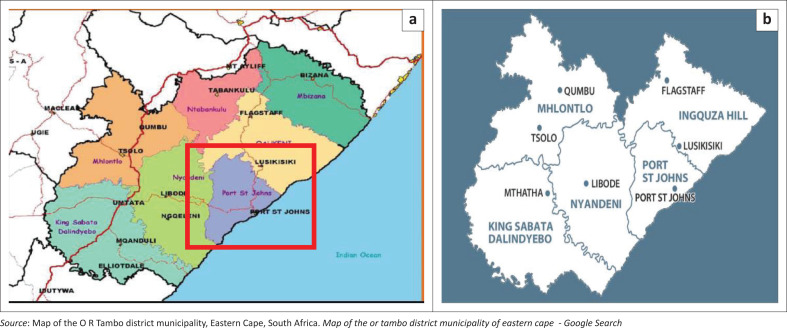
Google map of the (a) OR Tambo municipality, (b) Zoom in on red box.

The OR Tambo district has a population density of 113.6 people per km^2^ and 1 374 092 residents.^51^ Farming and trading are the occupations for the majority of the population.^51^ Over 90% of the population uses the public health sector for care.^51^ The district had a high TB incidence of 571 cases per 100 000 populations per year compared to a national average of 520 in 2015.^51^ It had lower rates of RR-TB confirmed client rate of 5.4% compared to a national average of 6.1%.^51^ Also, treatment success (39%) had lagged behind the national target (55%).^51^ The study was conducted at four treatment sites (Mthatha, Flagstaff, Bambisana and Libode) in four of the five sub-districts of the OR Tambo district municipality of Eastern Cape in April 2020 (Figure 1).^52^ The sites are mostly suburban and rural with only Mthatha being urban.

### Selection and recruitment of participants

The study population was defined as all patients with DR-TB. The initial sample size was 12 patients and 12 family members, although data saturation determined the actual sample size. The sample was said to be saturated when no new theme or information emerged during the course of the last three interviews.^16^ The attending nurse identified those patients who could articulate their views about community DR-TB management based on their ongoing interactions with the patients. Hence, a purposive sampling method that ensured those selected had characteristics of interest was used. In addition, patients who participated in the interviews were each requested to identify a family (household) member who had been available and supportive of them in the course of their illness, and willing to talk to the researcher about their experience of caring for patients with DR-TB.

### Data gathering method

An interview guide was developed with topics from the policy guideline, literature review, and inputs from health care workers (HCWs) working at the DR-TB units. The guide explored factors associated with diagnosis, treatment initiation, retention in care, loss to follow up (LTFU), and treatment outcomes. It also looked into misconceptions about DR-TB, the difficulties experienced, issues of stigma and coping strategies. Participants’ satisfaction and quality of care were assessed using a Likert scale of 1 to 10. The interview guide was piloted among two patients and two family members for completeness and appropriateness by the researcher and research assistant before they were handed to the participants a week prior to interview for them to reflect on the questions. The pilot interviews were not included in this analysis.

### Data gathering process

Participants (patients and families) were interviewed in the comfort of their community environment (at their homes or health centre) in order to allay any anxiety and inducement that may arise from the influence of a strange health care environment and HCWs.^16^ Interviews took place at the following four sites: Mthatha, Flagstaff, Bambisana, and Libode.

Two patients with DR-TB, who preferred to speak English were interviewed by the researcher and a trained research assistant interviewed 13 patients and all family members in *isiXhosa*.

All interviews were conducted in person and audiotaped. An open-ended interviewing style was used to elicit information from participants who were encouraged to speak openly and freely on the questions asked^17^ in order to uncover their own ‘framework of meanings’.^17^ Each of the interviews took between 15 and 23 min to be completed. Though the interviews were participant driven, the interview guide was used to keep the participants on track to avoid unnecessary information that may be irrelevant to the topic under discussion.

Depending on who was conducting the interviews, the researcher or the trained research assistant took turns to observe for non-verbal cues (facial expressions, nuances, and other body language, etc.) from the participants during the course of the interviews and made field notes. The researcher and research assistant were not involved in the medical management of the participants.

Transportation cost in rand (R40–R120) for attending the interview was reimbursed, and refreshment was served.

All interviews were transcribed and translated verbatim by the research assistant. An independent translator (a language teacher) fluent in both English and isiXhosa listened to portions of audiotaped interviews and then compared with the translated and transcribed data for accuracy and completeness in order to enhance the credibility of the data.

### Data analysis

All transcripts were used for the analysis. A framework analytic approach to qualitative data analysis with thematic content analysis was used in addition to the ATLAS.ti software programme. The framework analysis is an approach used to analyse and interpret narrative data,^18^ which is targeted towards a particular issue. The narrative data were transcripts from individual interviews as well as field notes obtained during the interviews. The process of analysis using the framework method involved:

- Familiarisation: In order to get acquainted with the data, the researcher read and re-read the transcripts and listened to the audiotaped interviews which allowed for organisation and preparation for the subsequent steps of analysis.
- Identification of the coding (thematic) framework: All issues identified during familiarisation were used to create a set of defined codes. Related codes were grouped together in categories.
- Indexing: A systematic application of the coding framework was performed across all transcripts.
- Charting: Codes were organised into code groups or families, and all data belonging to that group were brought together in the same report or chart.
- Mapping and interpretation: The data were interpreted to identify themes and subthemes as well as relationships or associations between themes^18,19^ and explanations were sought: explicit or implicit.

### Trustworthiness

The researcher employed the following strategies to improve the scientific rigour and credibility of this qualitative research.

#### Triangulation

This referred to the use of various data collection methods (interviews, observations and field notes) which helped to enhance both the credibility and comprehensiveness of the research as one method complemented the other. Congruency between the methods reflected the degree of agreement between the findings from both methods after comparison.

#### Member checking

To ensure the transcripts were accurate reflections of the interviews, each participant received a copy of their transcript to examine if their views were adequately captured. This allowed for clarifications and corrections.^20^ It also provided an opportunity to offer fresh views or additional information where necessary. In all, only one patient expressed concern regarding what was captured on her response to the use of traditional medications along with DR-TB treatment. This process of iteration further helped to strengthen the accuracy of the transcriptions.

#### Reflexivity report

A conscious awareness of reflexivity report that expressed the researcher’s own predispositions, biases, assumptions, understanding and epistemology was kept to maintain neutrality in the research process, and elucidate the lenses through which data were gathered, viewed and analysed (see Annexure 2 for a copy of the report).

#### Deviant case analysis

All data were given due consideration, and care was taken to ensure that ideas that appeared divergent from others or the overall theme were identified and included.

#### Fair dealing

All interview transcripts were analysed to elicit their views, and care was taken to ensure that no particular view or views were considered at the detriment of others during the interpretation phase. This process was also supervised by my supervisor as a form of peer review.

### Ethical considerations

Ethical clearance was granted by the Health Research Ethics Committee (HREC) of the Stellenbosch University (HREC Reference #: S18/01/013). The Eastern Cape Department of Health and the OR Tambo district municipality gave permission to carry out the study. All the participants interviewed in this study received a copy of the participant information leaflet written both in English, and the local language (isiXhosa) to go through before they signed the informed consent form. Confidentiality of information during and after the study is guaranteed. The study followed the Helsinki Declaration and Good Clinical Practice Guidelines.

## Results

A total of 30 persons were interviewed: 15 each of patients and family members respectively in pairs, but separately interviewed (patient first, then family member) (see Table 1 for the profile of the interviewees). For clarity, the results are represented separately below.

**TABLE 1:** Shows the profile of the patients and family members who participated in the interview.

Participants (patients & family members)	Age (years)	Gender	HIV Status	Occupation	Relationship to patient
P1	28	Male	-ve	Part time worker	Aunt
FM1	60	Female		Old age Grant	
P2	32	Female	+ve	Unemployed	Neighbour
FM2	47	Female		Trader	
P3	18	Female	-ve	Student	Mother
FM3	51	Female		Traditional healer	
P4	26	Male	+ve	Unemployed	Aunt
FM4	38	Female		Unemployed	
P5	67	Male	-ve	Pensioner	Wife
FM5	60	Female		Pensioner	
P6	42	Female	-ve	Teacher	Daughter
FM6	20	Female		Unemployed	
P7	18	Female	-ve	Student	Mother
FM7	50	Female		Trader	
P8	28	Male	-ve	Unemployed	Grandmother
FM8	68	Female		Pensioner	
P9	61	Female	-ve	Pensioner	Daughter
FM9	34	Female		Nurse	
P10	24	Male	+ve	Unemployed	Mother
FM10	57	Female		Teacher	
P11	28	Male	+ve	Social grant	Mother
FM11	52	Female		Trader	
P12	26	Male	+ve	Unemployed	Mother
FM12	45	Female		Unemployed	
P13	30	Female	+ve	Social grant	Mother
FM13	56	Female		Traditional healer	
P14	33	Female	+ve	Unemployed	Sister
FM14	49	Female		Business	
P15	46	Male	+ve	Unemployed	Wife
FM15	34	Female		Sales representative	

P, Patient; FM, Family member; +ve, positive; -ve, Negative.

## Perceptions of patients about the decentralised community Drug Resistant Tuberculosis care model

Overall, the semi-structured interviews conducted included 15 patients with DR-TB. This section provides the patients’ perceptions and experiences of the decentralised community DR-TB care model in the OR Tambo district municipality. It focussed on highlighting their knowledge, understanding of their illness experiences of DR-TB, and patient-related as well as health sector-related factors that could influence the effectiveness of the care model. The various interview questions as well as information provided by the participants form the basis of the themes. In all, 4 main themes and 12 subthemes emerged from this section.

### Theme 1: Knowledge of Drug Resistant Tuberculosis, its route of transmission and treatment

#### Theme 1.1: General knowledge of Drug Resistant Tuberculosis and its mode of transmission

The participants’ knowledge of DR-TB was satisfactory as only three had no knowledge of it. The rest of the participants explained what DR-TB is and how it is transmitted in their own words. None of the participants explained exactly what DR-TB stood for, but most of them could describe how it came about and that it is a ‘bigger’ form of TB. In their description, they also expressed the mode of transmission namely airborne or when you come in contact with someone who is coughing or sneezing.

Three of the participants understood correctly what DR-TB is, precisely stating that it is a drug-resistant form of TB which is transmitted via the airborne route following a cough or sneeze from an infected person. The representative quotes below reflect this further.

‘It’s a big TB, and it is transmitted by not taking medication properly because I was given treatment and I didn’t take them properly and I defaulted then I got this TB. You can also get it when you are around someone who has MDR-TB.’ (Participant 8)

‘According to my knowledge MDR-TB is tuberculosis that is resistant to medications; it can be transmitted by being around someone who has it.’ (Participant 12)

Nine of the participants understood DR-TB as a ‘big TB’, a disease of the lungs that is transmitted through airborne route or direct contact. The excerpts below highlight this further.

‘It is disease of the lungs. You get infected when somebody with the disease cough or sneeze around people without wearing a mask.’ (Participant 4)

Three participants were ignorant of what DR-TB is, and one of them had no understanding of how it is transmitted. The representative quotes below highlight this further.

‘I don’t know but mom said its dirtiness in your chest. I don’t know how its spread’ (Participant 10)

‘I don’t know, it gets transmitted through talking, laughing and singing when you’re next to someone and you have MDR-TB’. (Participant 11)

#### Theme 1.2: Satisfactory knowledge of side effects of DR-TB drugs enhanced adherence and prevented treatment defaults

All the participants had side effects within the first few days following treatment initiation. According to the participants, the most common problems they experienced with the DR-TB treatment were vomiting, itching, joint pains, body weakness, sweating, and diarrhoea. The excerpts below highlight this further.

‘I usually feel itchy then my joint becomes painful. Now I have stomach problems and I am losing weight and I no longer want to eat because I know I will vomit.’ (Participant 12)

‘All the joints are painful; my entire body is in pain and find it difficult to even sit down because my knees hurt. Also, body itch, diarrhoea, and sweating’ (Participant 8)

Patients’ knowledge of side effects of DR-TB drugs following adherence counselling strengthened their resolve to complete the regimen despite the unpleasant side effects experienced during the initiation phase of treatment. The representative quotes below highlight this further.

‘The pills are making me sick, but I endure them because they are helpful.’ (Participant 5)

#### Theme 1.3: Improved health status while taking Drug Resistant Tuberculosis treatment amid side effects

Participants experienced improvement in health status as symptoms subsided while on treatment. Some of them spoke of side effects at some point during treatment including vomiting, fatigue, pain and skin discolouration. Two participants discussed positive experiences of taking DR-TB treatment and specified improvement in health as sideeffects progressively abated:

‘When I started taking pills I saw the improvement, it was easy to breathe, I used to travel hundred metres (100 m) and rest, maybe resting ten (10) times walking uphill. I am no longer sweating even when sleeping.’ (Participant 4)

Three of the participants reflected on the side effects they experienced during the treatment. Vomiting, diarrhoea, body pains, and skin changes were some of the side effects the patients had:

‘Painful neck and legs are experienced when taking this treatment. You need to rest after taking pills and do not sleep until it is finally swallowed.’ (Participant 5)

‘I had diarrhoea and skin rash.’ (Participant 6)

‘My complexion turned black, and I lost weight.’ (Participant 10)

‘Skin complexion changes, I had spots nurses told me it is pills I will be right/fine.’ (Participant 13)

‘I don’t cough anymore. I am now able to eat. I couldn’t eat before, and my weight is back to normal, but my skin tone has change.’ (Participant 11)

### Theme 2: Patient help-seeking behaviour and reaction to the diagnosis of Drug Resistant Tuberculosis

#### Theme 2.1: Satisfactory help-seeking behaviour: Early presentation following onset of symptoms

The health-seeking behaviour of the participants was impressive as most patients sought medical intervention within a short space of time following onset of symptoms. Sputum presentation to establish a diagnosis was rapidly done and treatment was initiated immediately after confirmation of DR-TB.

Timely presentation following onset of symptoms could decrease onward transmission and lead to good patient outcomes. A good number of the study participants presented within a week or after a few days of experiencing symptoms:

‘I got sick for one day only and I went to the clinic.’ (Participant 9)

‘I finished one week before going to the clinic.’ (Participant 13)

Two participants presented after a month of experiencing symptoms:

‘I was sick for a month and was staying with my grandmother. I knew it’s because I defaulted the treatment for that TB.’ (Participant 8)

However, five participants reported delays of up to a month or more prior to presentation. These patients either presented to the clinic late, or were misdiagnosed initially:

‘About 3 months. It was long because I would be taken to the clinic and they would say I have fever until they made me cough (collected sputum), about 3 months.’ (Participant 10)

#### Theme 2.2: Patients suffered emotional difficulties when informed about a diagnosis of Drug Resistant Tuberculosis

Thirteen participants suffered significant emotional difficulties when first diagnosed with DR-TB. The most common emotion expressed was fear of death. Others expressed sadness, hurt, shock, and had low self-confidence. Their greatest concern following diagnosis was limited knowledge of the disease:

‘I cough then after I experience difficult breathing then loose energy. I get hurt because I know I have a disease and I do not understand the nature of the diseases I have. The way I understood the disease is that it’s going to kill me because I lost too much weight.’ (Participant 1)

‘I felt low self-confidence; I thought I am going to die.’ (Participant 7)

‘I was not happy at all because I once had TB, now I was amazed that how do I get it again because it was treated before and now it comes back as MDR. My worry was the pills because I knew they are a lot. I thought I am going to die because when I started treatment, I got sicker, after that I became better.’ (Participant 2)

Others were confident that they will survive the condition as a result of prior experience with a favourable outcome:

‘I was not concerned because my aunt had the disease and survived, so I knew I am going to survive.’ (Participant 4)

‘I didn’t think much of it because my grandmother also had it and she took her treatment then got better.’ (Participant 8)

### Theme 3: Long travel distances and transportation costs challenged access to treatment

Access to the clinic mirrored through the distance to the health facility estimated by transportation cost and the number of clinic visits made per month revealed that the participants spent approximately USD 4.50 (R 76.57) with an average of 1.3 visits per month.

#### Theme 3.1: Difficult and challenging access

The distance of the care centres from the residences of the majority of the participants (10 patients) presented a challenge to accessing care. Also, having more than one clinic visit per month (USD 6 [R 102.67]and 1.3 visits per month) negatively impacted access. Inaccessibility could hamper the effectiveness of the care model:

‘It is very far to the hospital, my clinic is the closest, I use R100 per visit.’ (Participant 2)

‘Not accessible, I use R100 and I come 2 times a month.’ (Participant 6)

‘It’s not accessible because it is far. I take a taxi for R30 to the stop then R25 to Mthatha then another R28 to get here. I come once a month.’ (Participant 8)’

‘It’s not affordable, I use R240 to come, I come once a month.’ (Participant 9)

#### Theme 3.2: Easy access to care centre

Few (five) patients had easy access to care and spent on average USD 1.73 (R 29.60) in transportation cost with most of them having a single clinic visit per month:

‘It is accessible, I use R20 and I come only once.’ (Participant 4)

‘It’s affordable. I use R10 to come and R10 to go home. I come twice a month when I come to get pill and when I come get.’ (Participant 11)

‘It’s accessible; I use R42 coming and going back. I come all the time when given visit date I will never make a mistake because these pills help me a lot.’ (Participant 13)

### Theme 4: A ‘ready’ health system influenced the effectiveness of community Drug Resistant Tuberculosis management

#### Theme 4.1: Rapid diagnosis after sputum presentation

Turn-around time from specimen collection to diagnosis was short ranging from same day to a maximum of 10 days:

‘I was made to cough and was told that I have MDR-TB same day.’ (Participants 5 and 10)

‘When I went to the clinic they said I have MDR-TB that day.’ (Participant 15)

#### Theme 4.2: Prompt treatment initiation after diagnosis

Early diagnosis and prompt treatment initiation are other components of an effective care model. Treatment was started immediately after diagnosis of DR-TB and no participant reported a delayed treatment initiation following diagnosis:

‘On the day I went to clinic they took sputum, after a day they called me to come back for results, I was told I have MDR, then they gave me pills (within 2 days).’ (Participant 1)

‘On the day of diagnosis in hospital I was given pills and took them same day.’ (Participant 2)

‘I started on the day I came here where I was told I have the disease.’ (Participant 13)

#### Theme 4.3: Patients received comprehensive care

The nurses provided the necessary support the patients required to facilitate their recovery. Drugs and free food parcels were also always available and given to most of the participants. In addition, five of them received financial support (social grant) made available to persons with DR-TB:

‘Nurses pay attention to us enough. I do have a grant that I already get, food, and I get the pills appropriately as well as support.’ (Participant 11)

‘Nurses take care of us a lot, they even phone us to come and get food because we are taking pills. I have social grant I get it.’ (Participant 13)

‘Nurses pay attention to us sufficiently. I was always getting grant money for the elderly, and I will start getting food from today. I get medication correctly.’ (Participant 9)

But some do not get financial support because of certain factors.

‘The nurses are very caring a lot. I don’t get grant because of my ID document. I do get food and medications. I also get support.’ (Participant 12)

‘I do get support they call sometimes to remind me, sometimes I do not take transport they fetch me since I have leg problem. I do get food but no social grant because I have a problem of ID document.’ (Participant 14)

#### Theme 4.4: Lack of continuity, integration and coordination of care

Except for two patients who were visiting the facility for the first time, 13 patients were not seen by the same HCWs or nurses at every clinic visit. They all noted that HCWs alternate duties or run shifts:

‘No, they alternate. Sometimes, you are seen by one nurse but at another time by a different nurse. But they all work together’ (Participant 8)

‘They rotate/take turns to be on duty.’ (Participant 13)

Patients did not get all treatment in one place. Of the six patients who had comorbidities, five were HIV positive and one had type two diabetes mellitus (T2DM). Among this group, only one patient received all treatments at the same clinic. Others do not:

‘I have HIV pills; I get all my medication here.’ (Participant 15)

‘No, the MDR ones I get them here in hospital. The HIV ones I get them from clinic.’ (Participant 2)

‘I am diabetic. I take diabetic treatment from specialist.’ (Participant 6)

#### Theme 4.5: Quality of care was closely associated with patients’ satisfaction

**Sub-theme 4.5.1 Excellent quality of care:** The majority (11) of the patients rated the quality of care received as excellent, giving a score of 10. Their reasons were that they were well treated, well taken care of, and never ill-treated:

‘10; I haven’t received an ill-treatment I am treated well.’ (Participant 9)

‘10; Because they call to remind me of my clinic appointment date. They also go to my home and check on me and people I stay with. I am happy and I take my treatment.’ (Participant 12)

Two participants rate the quality of care at 9. They specified the following:

‘9; They explain well when to comeback and what to eat, there are things that you should not do, you must wear warm cloths, wear mask when you are with people.’ (Participant 3)

Another two rated at 8, and their reasons were:

‘8; because from clinic I used my money for transport, but when I got here I heard there is an ambulance that picks people up.’ (Participant 2)

‘8; I am well taken care of when I come here.’ (Participant 4)

**Sub-theme 4.5.2: Patients satisfaction with the care provided:** Majority of the participants (12) indicated that they were extremely satisfied because they were well treated. However, few of them recommended the probability of staying at the facility until treatment completion to maintain adherence. Others expressed challenges including long distances and financial constraints for transportation to the facility:

‘10; I am satisfied. I wish there was a place where someone can stay in till they get better so they don’t infect other people and so I can recover, some people stop drinking medication and go back to their old routine as soon as they get back home.’ (Participant 11)

‘10; I am satisfied. I wish when they come they could bring treatment with them because this place is far.’ (Participant 12)

‘10; I am satisfied a lot I even see myself I am getting healed/ treated. In me everything is going well except that I worry because I have to borrow money for me to get here.’ (Participant 13)

One participant each rated their level of satisfaction at eight and nine and their reasons were:

‘9; I thought I will default along the way because treatment is too much. By being motivated here in hospital I got encouraged to not default.’ (Participant 3)

‘8; It is very far from my home.’ (Participant 6)

## Perceptions of family members about decentralised community Drug Resistant Tuberculosis care model in the or Tambo district

This section yielded 5 themes and 9 subthemes as reported below.

### Theme 1: General views and misconceptions about community Drug Resistant Tuberculosis care among family members

A common belief among family and community members about DR-TB is that it is a highly infectious and deadly disease. They perceived it as infectious because of the number of persons infected in the community. Surprisingly, it was associated with witchcraft because, in one household, all members were infected. Because of the community’s misconception about the disease, they tended to isolate themselves from and are afraid of patients with DR-TB. Another misconception was that only people infected with HIV contract DR-TB. A common perception about DR-TB is that it is a disease of the poor and unemployed because some people intentionally infect themselves to get financial support from the government:

‘They regarded it as a deadly disease that you must not be around people when you have it. Some people say it is a disease of poverty because people deliberately infect themselves because they want money.’ (FMP 5)

‘In the community no one comes to check on him not even his friends, He is isolated.’ (FMP 12)

‘People think it is the big TB, they think people who have this TB is one who is HIV positive.’ (FMP 14)

‘My sister once had so it was not the first time to hear about the disease, but when experienced I thought it is witchcraft because we were 5 at home with MDR.’ (FMP 4)

### Theme 2: Patients’ experiences from sputum production for testing through diagnosis to treatment

#### Theme 2.1: Rapid diagnosis following sputum production

Nearly all the family members said diagnosis using this model of care was timely. Most of the diagnoses were confirmed within the same week of sputum collection. The contact family members were notified of the outcome of the results as soon as they were available:

‘They took sputum, was called following day to come and collect results.’ (FMP 3)

‘Sputum was collected, after 3 days I was called to collect results.’ (FMP 6)

‘There were days that passed before he got results. They made him cough then we went home, they called to tell him the results.’ (FMP 11)

#### Theme 2.2: Treatment counselling and adherence

The majority of the family members confirmed being counselled by the HCWs on treatment and adherence. Drug Resistant Tuberculosis was explained as well as its side effects and prognosis:

‘Yes. They said he has big TB MDR. They said it is treatable my God! and the person who has gets treated when he takes pills the way they were instructed.’ (FMP 1)

‘Yes; we were told we need to stay safe because it is highly infectious and we need to pay attention to the child and make sure they take their pills appropriately.’ (FMP 15)

One participant was however dissatisfied with the counselling provided while two others said they received neither treatment nor adherence counselling:

‘Yes, but we were not satisfied about how it’s was explained how it’s like.’ (FMP 9)

‘No, it was never explained.’ (FMP 3)

### Theme 3: Travel distance and cost of transportation to health facility

The distance between the health facility and the patient’s residence as well as other factors such as the terrain, and availability of good roads determined the cost of transportation. Transportation cost was exorbitant for more than half (9; 60%) of the participants. Some had to borrow money from neighbours while others hired a car to get to the facility:

‘It’s far and isn’t accessible! We have a challenge in term of finances, I go with R126 and I will come back with another R126.’ (FMP 9)

‘It’s not affordable because no one works so I have to borrow money so I can take care of my child’s health.’ (FMP 12)

Only 4 (26.7%) participants stated that the health facility was accessible. The minimum and maximum transportation cost were R 20 and R 40 respectively:

‘It is accessible because we use R40 to and fro.’ (FMP 4 and FMP 5)

‘It’s affordable, I use R20 to go and I only go when a parent is needed.’ (FMP 11)

### Theme 4: Activated healthcare workers (well-trained, knowledgeable and ready) with positive attitude, short waiting time, and good quality of care resulted in patients and family’s satisfaction

#### Theme 4.1: Waiting time at the health facility

There were no delays on drug collection days. The waiting times ranged from 10 min to an hour:

‘Ten minutes.’ (FMP 14)

‘Not long. They care a lot, what I like is that you come once and when you return you are already known, they know everyone by name.’ (FMP 10)

‘It depends on how many people are here when you get here, I would say it’s usually 30 minutes to an hour.’ (FMP 9)

#### Theme 4.2: The attitude of healthcare workers

The participants noted that the HCWs at the clinics were friendly and genuinely concerned about the care of the patients:

‘Nurses take care of us and they are very friendly.’ (FMP 7)

‘I am actually impressed but he is the one who has trouble stopping drugs.’ (FMP 11)

‘Nurses are taking care of us. They have good sense of humour.’ (FMP 13)

One of the participants expressed that not all the HCWs were friendly with the patients:

‘They are not the same, some are friendly.’ (FMP 3)

#### Theme 4.3: Quality of care

Majority of participants rated the quality of care received as excellent, giving a score of 10/10. Although none of them rated the quality as poor, a participant each gave a score of 9 and 6, respectively:

‘10; because I am well taken care of and they are efficient’ (FMP 7)

‘10. They are alright, they explain everything clearly.’ (FMP 15)

‘9. They are right/good they explain everything. Nothing lacks except that it is too far.’ (FMP 14)

‘6. They do not care much, sometimes you arrive early, and they also arrive early but not help us. They start at 11am to help us.’ (FMP 3)

#### Theme 4.4: Relatives’ satisfaction

Nine of the relatives were very satisfied with the services received and rated their satisfaction as 10/10. Two participants scaled theirs at 9, one at 8, and another at 7:

‘10. If he didn’t come here at gateway may be he would have died. I am very satisfied.’ (FMP 12)

‘10. they are taking care they phone to remind you of the appointment date and also check if you did not come.’ (FMP 14)

‘7. I am very much satisfied. There is nothing short except the need for transport because it is far here.’ (FMP 15)

One participant was satisfied but did not rate the level of satisfaction:

‘I see help here, where you see help it is the place you really need to be at.’ (FMP 13)

### Theme 5: Views about traditional medicines and traditional healers regarding Drug Resistant Tuberculosis

#### Theme 5.1: General views on reliability of traditional medicines and Drug Resistant Tuberculosis

Full trust in medical advice and pharmaceutical (medicine) was evident in the responses of 13 participants. This trust stemmed from a strong belief that traditional medicine cannot treat DR-TB, traditional healers do not have the necessary facilities, and that medicines should not be mixed:

‘I do not think traditional medicine can work in MDR, because it is worse than normal TB. You need to endure pills.’ (FMP 4)

‘Traditional medicine and healers can never treat this TB because they do not have resources that hospital have.’ (FMP 7)

‘I don’t agree with traditional medicine because they are not 100% and they can’t tell us about their side effects.’ (FMP 9)

‘I don’t recommend traditional medicine because its medication that is not properly researched about this disease so I recommend help from the clinic.’ (FMP 11)

Only two participants believed they could use both traditional and modern medicines concomitantly:

‘It can be used.’ (FMP 3)

‘As you can see I am a traditional healer and wearing beads and traditional healer things. I use traditional medicine when things get out of hand.’ (FMP 13)

#### Theme 5.2: Consultation with traditional healers

Nearly all of the participants did not consult a traditional healer on behalf of their relatives with DR-TB:

‘No; I did not want to send him to traditional healer because I saw that clinic is the one that will be able to diagnose.’ (FMP 7)

‘No I didn’t take her to a traditional healer she needs to go to the clinic because the healer might not see what is wrong.’ (FMP 8)

Only one of the participants consulted a traditional healer but was advised to seek medical intervention:

‘Because of not understanding him when collapsing I took him to traditional healer, then traditional healer said we must take him to clinic because some things need clinic.’ (FMP 13)

#### Theme 5.3: Combined use of clinic and traditional healer’s treatment

Concomitant use of both DR-TB drugs provided by the health facility and traditional medicine was not practised by any of the participants. Even those participants who used traditional medicine reportedly stopped when their relative commenced the DR-TB drugs given by the health facility:

‘No. Medicines are not to be mixed; if you are using ones from hospital you should not use traditional medicine.’ (FMP 2)

‘I think it is not good to mix them because a person with TB is weak and traditional medicine is strong it needs strong person so that interrupts medication.’ (FMP 14)

## Discussion

The evaluation of the decentralised community DR-TB care model revealed a scaled up availability with five sites in the OR Tambo district, and it was largely acceptable. Patients felt at home and expressed satisfaction with the quality of care that allowed for a focus on all aspects of DR-TB management in the friendly environment and comfort of their communities.^21,22^ In-patient cost is reduced or eliminated,^23^ and the potential for nosocomial transmission of DR-TB is also reduced^24^ while allowing for patients’ participation in social activities such as work and family.

**FIGURE 2:**
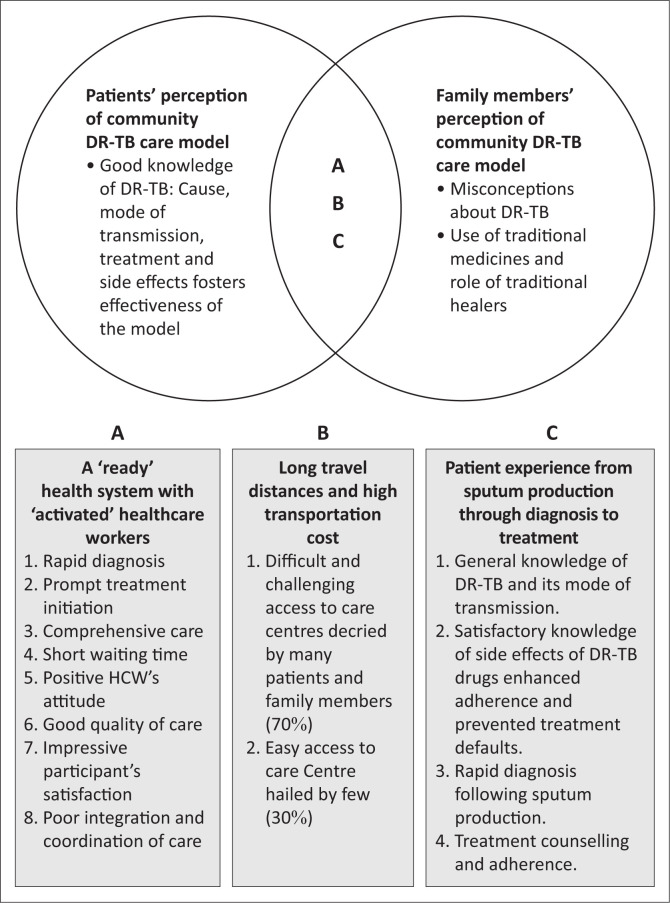
Conceptual framework of the results: Showing interactions between the overlapping perceptions of patients and family members on the effectiveness of a community DR-TB care model.

Figure 2 shows the relationship between the overlapping perceptions of patients and family members. Both shared some misconceptions about DR-TB including its cause and potential sufferers. Some attributed DR-TB to witchcraft as in certain instances, entire households were affected. Though it is true that people living with HIV are particularly vulnerable to contracting DR-TB,^25^ not all patients with DR-TB are HIV-infected or have comorbidity. Drug Resistant Tuberculosis without effective treatment is associated with high mortality, especially among those with comorbidities such as HIV and diabetes mellitus (DM).^26,27^ The advent of modern technologies for rapid diagnosis coupled with prompt initiation of effective treatment has, however, started reversing this narrative. Outcomes of DR-TB treatment have been improving incrementally from 39% to 50% to 60% or more over the years.^1,14,28^

Rapid diagnosis among this cohort using molecular tests such as Xpert MTB/Rif assay and the line probe assay (LPA) allowed for results to be available in real time (either same day or the following day). South Africa is among the first 20 of the 30 TB high burden countries that reached over 80% DR-TB testing rate.^5^ Treatment initiation appeared to have gone according to the recommendation by the health authorities (within five working days)^29^ as many participants reported being diagnosed and initiated treatment promptly. The ‘FAST’ approach to care means finding cases actively through cough surveillance and rapid molecular testing, separate safely, and rapidly treat based on DST.^30^ This approach to DR-TB care renders patients rapidly non-infectious, thereby halting potential transmission within households and the community at large.^31^

Most patients had a good knowledge of DR-TB and its transmission. Family members were quite convinced that only orthodox treatment could cure DR-TB as they claimed that traditional medicine would be too strong for their weak relatives. They complained about the doses and side effects of traditional medicines that are improperly researched or unknown. They also opined that traditional and clinic medicines should not be used concurrently. This understanding of supposedly negative impact of traditional medicine on patients’ health could be particularly rewarding as the potential for unnecessary interactions, either drug-drug or drug-disease interactions, is eliminated.

Contrary to findings by another study,^32^ HCWs in this study setting were knowledgeable about the disease, well trained and equipped with the necessary skills (activated) required to deal with DR-TB. Support received by the participants from the healthcare sector included psychosocial (counselling), economic (grants), nutritional support (food package)^33^ and treatment education, thereby making the illness experience a positive one for most patients. Patients’ counselling at every visit helped them to cope with side effects resulting in a low treatment default and LTFU among the cohort. A study in India^23^ showed that psychosocial support strengthens adherence and prevent depression,^24^ and improve quality of life.^34^ The majority of the participants were petrified on learning about their diagnoses. Though fear of death, poor knowledge of the disease and high pill burden, amid a potentially looming stigmatisation were their immediate reactions, reassurance by HCWs and family support played a significant role in the care of patients in this model. Even after rejection by friends and colleagues, family was there for most patients.

Co-morbid conditions such as HIV and DM have traditionally been associated with DR-TB.^35^ Both of these conditions are known to cause immunosuppression in affected individuals thereby increasing their vulnerability towards contracting TB.^35^ A study^36^ reported that patients with DM were twice as likely to be affected by DR-TB compared to those without it.^36^ A cost effective management of comorbidities is of utmost importance.^11^ However, care coordination and integration remained challenging for patients with co-morbid conditions, contributing to the increased transportation cost as they visited the health facilities on different days for their comorbid conditions. The axiom of ‘one patient, one folder, and one HCW’ remained elusive for most patients despite its advantages of promoting efficiency within the health system in delivering cost effective services and providing opportunities for managing drug interactions and adverse reactions.^37,38^ For effectiveness, rather than using a ‘silo’ approach to patient care where each condition is managed vertically, a combined vertical and horizontal approach to patient management would be potentially more cost effective for both the patient and care providers. However, this may require re-organisation of the health system, a mind-set shift, and retraining or upskilling of HCWs.

Access to care was problematic for most of the participants as long travel distances, exorbitant transport fares and the frequency of clinic visits meant more expenses for both patients and relatives. The UHC target 3.8 of the Sustainable Development Goals (SDGs) advocated by the WHO^5^ is aimed at reducing cost and increasing access to care. However, UHC remained a mirage for most patients in this cohort and a participant even expressed preference for centralised model in order to cut transportation costs. Likewise, long treatment duration decried by some participants negatively impacts adherence owing to exorbitant transportation costs. The WHO now advocates an all-oral regimen that incorporates new and repurposed drugs such as bedaquiline, clofazimine, and linezolid,^25^ and reduces treatment duration to 6 months.^39^ Alternative mode of delivering medications such as the Central Chronic Medicines Dispensing and Distribution (CCMDD) system which utilises designated pick-up-points could bring care closer to the people and improve access especially to stable patients.

The neurotoxic and hepatotoxic potentials usually associated with DR-TB drugs contributed to the appearance of side effects reported by the patients.^14,35,40,41,42,43^ These included vomiting, itching, joint pains, body weakness, skin discolouration, sweating and diarrhoea. Adherence counselling and treatment support assisted patients to deal with them and prevented default.

Regarding community perception of DR-TB, most family members believed it is a ‘deadly disease’ hence, their first reaction to the diagnosis was that of fear of death and isolation. Drug Resistant Tuberculosis remained a feared and highly stigmatised disease within the community. This perception is changing as they witnessed patients’ survival and improving health status instead. One distinguishing factor that identified a patient with DR-TB in the community was the wearing of mask and one participant was very grateful to coronavirus disease 2019 (COVID-19) which ensured universal mask wearing and level playing field, although COVID-19 pandemic has had a negative impact on the TB programme overall.^5^ Progress made on expanding access to TB diagnosis and treatment in the years leading up to 2019 had slowed, stalled or reversed, and global TB targets are off track.^5^

Overall, satisfaction was closely linked to the quality of care received by both the patients and family members. They were pleased with the care given by the nurses. Some of the qualities expressed included simple courtesy (greeting and knowing everyone by name), counselling and encouragement, sending reminders (phone calls and messages) about treatment appointments and collection of food parcels. Satisfaction with care may be associated with adherence and favourable treatment outcome. This is similar to findings from other studies which reported that provision of psychosocial support, education and treatment literacy^44^ as well as shared decision-making are important towards achieving favourable outcomes.^45^ However, two participants decried receiving any counselling prior to treatment initiation.

Community DR-TB management is both patient, community, and HCW driven.^46^ Medications are self-administered though with support from family members and HCW, concurrent with findings from another study.^46^

Poor adherence was initially thought to be the driver of DR-TB until it was shown to be driven by primary transmission.^47,48^

Some of the advantages of the community care model include reduced LTFU,^13^ and better cure rate.^49^ Treatment success for 2019 cohort was 60% up from 50% in 2012.^1^

A holistic approach which addresses DR-TB beyond the healthcare system by focussing on other determinants of health such as poverty alleviation, provision of good roads, community education on the treatability and preventability of DR-TB need to be advocated as this has been found to reduce stigma and in turn influence care-seeking behaviour and adherence.^45^ The community DR-TB care model embraces most of the components of holistic care, hence its ‘perceived effectiveness’ in the OR Tambo communities. However, holistic care would be incomplete without palliative care as few people may benefit from some forms of relief or palliation at some point in time.^50^

## Limitations

This study is limited by the number of data gathering methods used (semi-structured interviews and field notes). A focus group of community members and patients could have complemented the information provided by the participants. Complemented with quantitative research, the full effectiveness of the community DR-TB care model could be unravelled.

## Recommendations

Though the community care model has been praised by patients and community members, transportation arrangements that meet the patients halfway will do so much good at improving the overall outlook of the model especially for those patients who require more than one stop to reach their care centres. Future research should look into what impact providing transportation at a central place where patients could gather and be transported to the clinic or get the treatment to the patients in their communities using local courier services such as motor-bike delivery system could make.

## Conclusion

Community DR-TB care model in the OR Tambo district municipality has been largely acceptable and perceived as effective in quality, timeliness and comprehensiveness of care by both patients and family members. However, poor coordination and integration of care along with limited access and affordability owing to long travel distances to care centres and high transportation cost remains challenging for most patients and family members.

## Annexure 1: Interview guide.

### Interview Guide

#### Preamble

The interview is part of a research project to obtain the views of HCWs on MDR-TB. There may be a delay between the time that sputum is taken for examination and the time that patients are started on MDR-TB treatment. This project aims to explore the views of HCWs on whether they think there is a delay and if so, the possible reasons for this, the implications of the delay, and what can be done about it.

### DEMOGRAPHICS

**Category of nurse/doctor:**

**Job Title/Position:**

**Age:**

**Gender:**

**Highest Educational Qualification: …………………………………………………………………………**

**(E.g. Matriculation, Diploma, Bachelor Degree, Masters, PhD, Certificate, Other)**

**No. Years working in current position (in CHC/Clinic/Di strict Office) ……………………………**.

### INTERVIEW GUIDE

#### Interview schedule (Patient)

1). **General knowledge of MDR-TB**

What is MDR-TB?

How is it transmitted?

What problems do people have with MDR-TB treatment?

2). **Patient related factors**

Tell me about your illness/sickness

Length of time before presentation: ‘**How long were you ill before you went to the Clinic/ GP/other healthcare provider to be diagnosed for MDR-TB?’**

How long it took to be diagnosed with MDR-TB:

Length of time between sputum production (presentation) and diagnosis: ‘**How long did it take for you to be diagnosed with MDR-TB after submitting sputum to the clinic?’**

How did you get started on treatment?

Length of time before treatment: **‘How long did it take to be started on treatment after you were diagnosed with MDR-TB?’** From diagnosis to treatment initiation should be 5 days. **If the response presumed any DELAY, ask why?**

Perceptions of danger to health & life/ complications; fear of death:

Reaction – individual: **‘when you were first t old that you had MDR-TB, how did you feel?’**

**‘What were your greatest concerns or worries about MDR-TB?’**

**‘What did you think it could do to you?’**

Experience of treatment and possible side effects – **‘What is your experience with taking the MDR pills?’**

1

#### Health Sector Factors

1). Access to care – freely accessible or not? ‘**What can you say about *ACCESS* to clinic, like for example: the *distance* between your home and the clinic, how much does it *cost* to and from clinic per visit?’**

**How many visits per month?**

2). Comprehensiveness of care – **‘What would you say about the total package of care you have received so far from the health care system?’** E.g.

**‘Are the nurses friendly and concerned about your health?’**

**‘ Do you get the necessary support from the health system?’**

3). Continuity, integration and coordination of care – **‘Are you seen by the same set of HCWs or nurses every time you attend clinic?’**

‘**Do you get all your treatment in one place?**

**if for instance, if you are on BP or Pressure treatment or ARVs or Diabetes treatment along with MDR-TB treatment at the same time and place?’**

4). **Efficiency of care**

Quality of care: **‘In terms of the *quality* of care you have received so far, on a scale of 1 to 10 where 1 is very poor care and 10 is excellent care, what would you rate the quality of care you have received?**

**and why have you chosen that figure?’**

Patient satisfaction: ‘what is your overall level of satisfaction with the care you have received so far?’ **on a scale of 1 to 10 where 1 is NOT SATISFIED and 10 is EXTREMELY SATISFIED and 5 is AVERAGE, what would you rate your *level of satisfaction* to be?**

2

### Discussion with family: attitudes & perceptions of family

#### Community Factors

Attitudes & perceptions of family, neighbours and community members

1. What do family members and the general community think about MDR-TB?

**‘Are these patients dirty?’?**

**‘Is it a disease of poor people?’**

HIV stigma – ‘**Will they be labelled as HIV if diagnosed with MDR-TB?’**

**‘ What do people associate with TB?’**

**‘What do they talk about with TB?’**

2. Diagnosis pathway

Tell us about your **patient experience** from producing sputum for testing to diagnosis? For example, **‘was the result available on the day the sputum was taken?** Or **did you have to come back for the result?’** Or

**were you called by the clinic staff that the result was ready? And when you came back was the results present or not?**

3. Treatment

In this segment, we want to know if any counselling was given by the HCW or NURSE and if so what were they counselled about?

**‘Was there any counselling by the Clinic Nurse and if YES, what were you counselled about?’**

(Adherence – understanding, Their own views)

Accessibility – distance, waiting times:

**In your own view, ‘what would you say about ACCESS to the Clinic in terms of distance and how much do you and your relative have to pay (cost) to get to the clinic?”**

**When you come to the clinic to get medications, how long must you wait before you are served?**

3

**Use of Traditional medicines and Traditional healers**

[Preface: Many people use traditional medicines]

Their Beliefs

Whom do they consult? [Herbalists, diviners ‘amagqira’, faith healers ‘abathandazeli’,

Do they use them first?

Do they use them together?

**‘What is your view about Traditional medicines and Traditional Healers regarding MDR-TB?”**

**‘Did you consult one regarding your relative and when?’**

**‘Do you use the treatment from CLINIC and TRADITIONAL HEALER together?’**

4). Attitudes of healthcare workers

**‘ What do you think about the Nurses at the clinic?’**

**‘Are they friendly and do they show genuine concern about the care of the patients?’**

5). Quality of care

Quality of care: **‘In terms of the *quality* of care you have received so far, on a scale of 1 to 10 where 1 is very poor care and 10 is excellent care, what would you rate the quality of care you have received?**

**and why have you chosen that figure?’**

6). Relative’s satisfaction:

‘what is your overall **level of satisfaction** with the care you have received so far?’ **on a scale of 1 to 10 where 1 is NOT SATISFIED and 10 is EXTREMELY SATISFIED and 5 is AVERAGE, what would you rate your *level of satisfaction* to be?**

## Annexure 2: Reflexivity report.

As a doctor with interest in TB, I have tried to carry out this research with an open mind being mindful to ensure that my knowledge of DR-TB was not superimposed on the views of the participants by allowing them to speak freely without interruptions. Notes were however taken during the interviews and clarifications were sought at the end of the interview regarding the information that was shared by the participants.

I recognised that my own values, beliefs, and assumptions may influence the research process. Likewise, I was also aware of the power dynamics that may have existed between the research participants and me. So, I strived to be aware of my own biases by ensuring that this research was conducted in a way that is ethical and respectful of their experiences, and being opened to new perspectives.

If I do have any prejudices or biases regarding DR-TB unknown to me, I have ensured that they are bracketed at all stages of this research process allowing for a free and fair deductions to be made from the data.
